# Presidential Address to Fulton County Medical Society

**Published:** 1916-01

**Authors:** W. A. Selman


### Journal-Record of Medicine

Successor to Atlanta Medical and Surgical Journal, Established 1855
and Southern Medical Record, Established 1870
**OWNED BY THE ATLANTA MEDICAL JOURNAL COMPANY**
PUBLISHED MONTHLY

*Official Organ Fulton County Medical Society, State Examing Board, Atlanta, Birmingham and Atlantic Railroad Surgeon's Association, Chattahoochee Valley Medical and Surgical Association, Etc.*

RICHARD R. DALY, M. D., Editor
BERNARD WOLFF, M. D., Supervising Editor
A. W. STIRLING, M. D., C. M., D. P. H.; J. S. HURT, B. Ph.. M.D.
GEO. M. NILES, M. D., W. J. LOVE, M. D., (Ala.); Associate Editors
E. W. ALLEN, Business Manager
**COLLABORATORS**
W. F. WESTMORELAND, M. D., General Surgery
F. W. McRAE, M. D., Abdominal Surgery
ALLEN H. BUNCE, Medical Societies
E. B. BLOCK, M. D., Diseases of the Nervous System
MICHAEL HOKE, M. D., Orthopedic Surgery
CYRUS W. STRICKLER, M. D., Legal Medicine and Medical Legislation
E. C. DAVIS, A. B., M. D., Obstetrics
E. G. JONES, A. B., M. D., Gynecology
ALLEN H. BUNCE, M. D., Pathology and Bacteriology
R. T. DORSEY, JR., B. S., M. D., Medicine
L. M. GAINES, A. B., M. D., Internal Medicine
GEO. C. MIZELL, M. D., Diseases of the Stomach and Intestines
L. B. CLARKE, M. D., Pediatrics
EDGAR PAULLIN, M. D., Opsonic Medicine
THEODORE TOEPEL, M. D., Mechano Therapy
R. R. DALY, M. D., Diseases of the Eye, Ear, Nose and Throat
BERNARD WOLFF, M. D., Diseases of the Skin
E. G. BALLENGER, M. D.. Diseases of the Genito-Urinary Organs

VOL. LXII. ATLANTA, GA., JANUARY, 1916. NO. 10

### PRESIDENTIAL ADDRESS TO FULTON COUNTY MEDICAL SOCIETY. TAKING STOCK.

W. A. SELMAN, M. D.

Now that we have a society of 260 members, what are we going to do with it? Soon the glare of the limelight of the whole stage of Southern medicine will be turned upon us, and shall we be found as 260 individuals, working as our separate inclinations dictate; or shall we be found as 260 members of a unified body, working together for the good of our society, our city, our country and ourselves!

If our lights are to shine abroad, they must first shine at home. Are we to try to see how near we can come to a record already made, or are we to establish a record that will be a standard of excellence for ensuing years? Let the latter be our aim! To do this will mean that every member should resolve to do his best and feel a sufficient interest in this society to come to its meetings and contribute to its success.

If only half of our members would attend meetings, no one would want for entertainment. Whenever one hundred Atlanta physicians get together, and cases are presented, or any paper read they will bring forth ample discussion from many points of view. We can boast of Surgeons, Ophthalmologists, Internists, Gynecologists, Pediatrists, Neurologists, Orthopaedists, Radiologists, Obstetricians, Bacteriologists, Urologists, Dermatologists, Anaesthetists, and Serologists. We look with pride upon these leaders in their respective specialties, and we need each other's help more and more as we learn that we cannot be masters of all subjects, but instead must be content to do a few things well.

We can boast of another class of physicians, the rank and file of our profession who say not, "I do this and this alone," but who go wherever duty calls them, and do what is in their power to relieve suffering in whatever form they find it. Allow me to pause here long enough to pay a deserved tribute to these followers of Aesculapius. Where else can you find 260 doctors where not one revels to excess at the court of Bacchus, not one dreams his hours away by casting his lot with the Lotus eaters, not one fails in his duty toward the needy, every one a gentleman?

There is scarcely a time during the year that some of our members are not visiting the centers of medical thought throughout the country. When the present European war broke out no less than six of our members were gleaning the medical fields abroad. These men come back to us and tell us many of the most recent achievements in medicine and surgery and give us up-to-date methods, months before we get them from Journals, and years before they appear in books. Indeed medical advances are so rapid that by the time many of our volumes are off the press, they are already being superceded by newer methods. This society is the mouthpiece of medical advancement, so do not read its program and say that there is nothing of interest to you, for on any evening may be presented clinical ideas that bear directly on your particular line of work and the discussion of which migth be invaluable to you. Indeed, it is the only post-graduate school we have. Here any one of us may bring our patients, exhibit them, demonstrate what we have done, or seek advise as to what is best to do.

Our scope has been enlarged, and we now have our Eclectic and Homeopathic members meeting with us, for they are a part of us. Let us learn of them—let them learn of us.

Let us all study Article II of our own Constitution—that of our State, and Americal Medical Associations. It will give us a broader conception of our duties and responsibilities. If we live up to them, we will not only love one another better, but be loved tenfold by those who look to us for help.

It becomes my duty to appoint certain committees for the ensuing year—our committee on Public Health and Legislation, have good laws to guide them. May they have no trouble in enforcing them! Our committee on Publication, I especially urge to activity. The public needs and demands education in medical matters. Shall we allow its mind satiated and distorted by extravagant misstatements of the lay press, or shall we give it conservative and sound advice on timely subjects, edited by our committee, censored by our censors, and backed by our society?

Let us burst the chrysalis of silence, and start a propoganda of medical education of the masses. What does it profit the public, for a physician to know the danger of an apparently benign tumor, when the patient consults him only after it is evidently malignant? If it takes joint meetings with the public to impress these truths, let us have an occasional meeting!

Our library has been a subject of much concern to me. I have tried to get the library habit. I have not succeeded, but am still trying. Those of us who have had the advantage of a medical library thus far in our medical education should stop and think what an advantage it would have been many times if we could have taken up a catalogued index, and read with profit the views of different authors on one subject, instead of consulting the views of one author in a text-book on many subjects. We all know a good medical library is a help to a community, and if there are those among us who have their own libraries and Journal files, or those who are too busy to use a library, remember there are others not so fortunate. Let us all contribute to its support, that we may enjoy the fruits of those who labor therein. Whatever plan for its future is recommended by our library committee, let us support it. Those interested in it will find it wherever it is, and those not interested would not use it, were it at their door.

I wish from my heart to thank each of you for the honor of being your President. I need your counsel and support. For you, I promise to do my best. I wish to salute my fellow officers and to promise them every assistance in my power.

Let us resolve, that whatever is done, we will all do it and wherever we meet, we will all be there!

